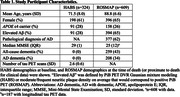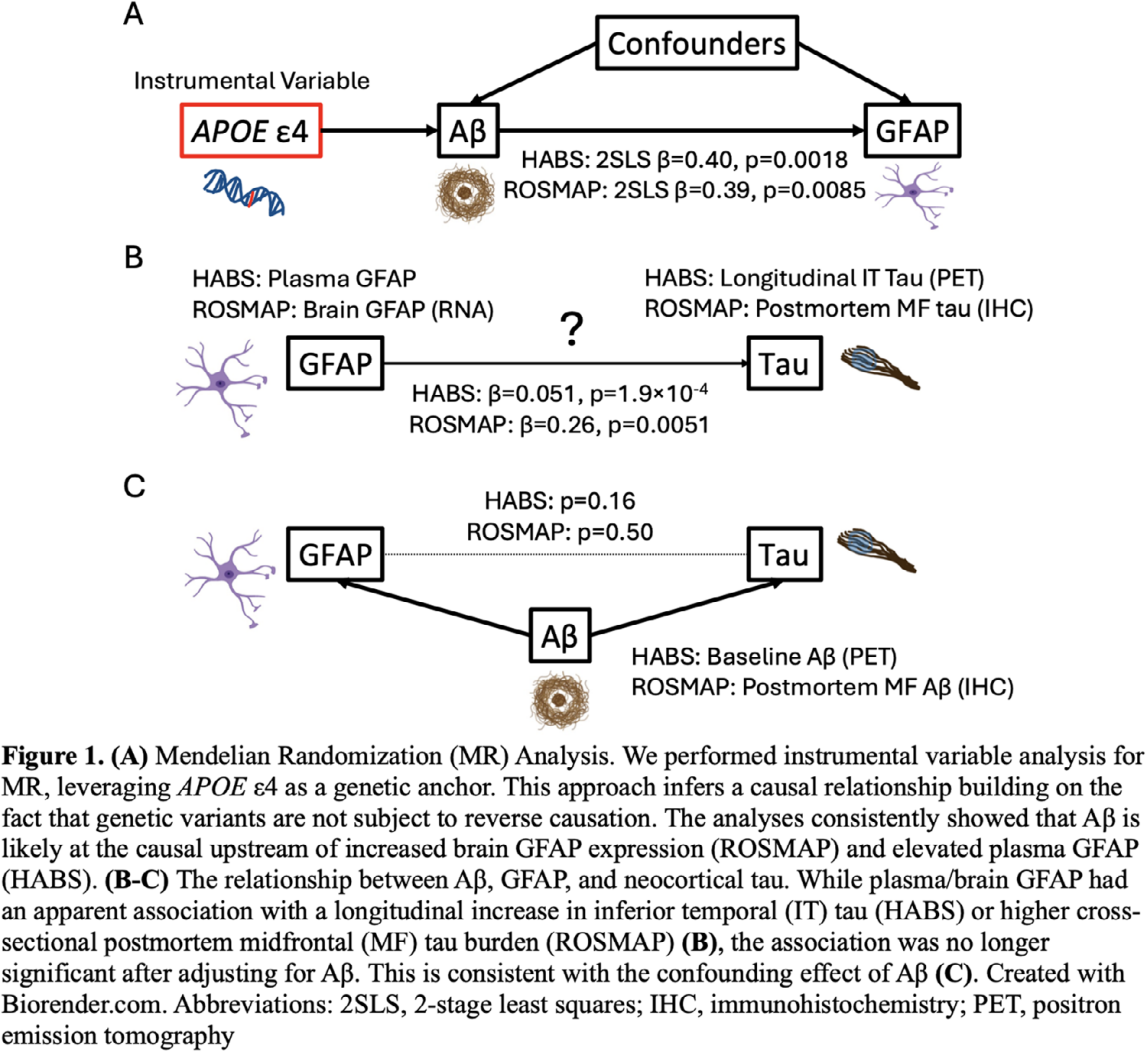# The relationship between amyloid‐β, plasma/brain GFAP, and neocortical tau: a Mendelian randomization study

**DOI:** 10.1002/alz.091415

**Published:** 2025-01-09

**Authors:** Hyun‐Sik Yang, Pia Kivisäkk, Andrea M. Román Viera, Courtney Maa, Dylan Kirn, Jean‐Pierre Bellier, Lei Liu, Dennis J. Selkoe, Julie A. Schneider, David A. Bennett, Philip L. De Jager, Keith A Johnson, Reisa A Sperling, Steven E. Arnold, Jasmeer P. Chhatwal

**Affiliations:** ^1^ Broad Institute of MIT and Harvard, Cambridge, MA USA; ^2^ Brigham and Women's Hospital, Boston, MA USA; ^3^ Massachusetts General Hospital, Boston, MA USA; ^4^ Harvard Medical School, Boston, MA USA; ^5^ Massachusetts General Hospital, Harvard Medical School, Boston, MA USA; ^6^ Rush University Medical Center, Chicago, IL USA; ^7^ Rush University, Chicago, IL USA; ^8^ Columbia University Irving Medical Center, New York, NY USA; ^9^ Columbia University, New York, NY USA; ^10^ Brigham and Women's Hospital, Harvard Medical School, Boston, MA USA

## Abstract

**Background:**

Plasma glial fibrillary acidic protein (GFAP) is associated with amyloid‐β (Aβ) and tau, but their causal relationships remain unclear. We used Mendelian randomization (MR; using genetic causal anchors to avoid reverse causation) to clarify the causal relationship between plasma/brain GFAP, Aβ, and tau.

**Methods:**

Study participants are from two independent datasets: the Harvard Aging Brain Study (HABS) and the Religious Orders Study/the Rush Memory and Aging Project (ROSMAP) (Table 1). From HABS, we used baseline plasma GFAP (MSD platform) and Aβ (PiB PET cortical composite with partial volume correction [PVC]) and longitudinal inferior temporal (IT) tau (FTP PET SUVR with PVC) data. From ROSMAP, we used post‐mortem frontal cortex GFAP expression (RNA‐Seq) and midfrontal Aβ and tau burden (immunohistochemistry). We used linear regressions to assess the relationship between APOE ε4, GFAP, and Aβ. For MR, we performed instrumental variable (IV) analyses using APOE ε4 as the instrument. In HABS, we used linear mixed effect models to assess baseline GFAP ‐ longitudinal IT tau association. In ROSMAP, we assessed the cross‐sectional GFAP ‐ tau association (linear regression). All variables were standardized, and all models were adjusted for age and sex.

**Result:**

In HABS, APOE ε4 carriers had higher baseline plasma GFAP (n=324, β=0.32, p=0.0025), but this association attenuated after adjusting for Aβ (p=0.42). MR suggested the causal effect of Aβ on plasma GFAP (2SLS β=0.40, p=0.0018). Baseline plasma GFAP predicted longitudinal increase in IT tau (n=187, β=0.051, p=1.9×10^‐4^); this association attenuated after adjusting baseline Aβ and its time‐interaction (p=0.16). We observed consistent results in ROSMAP: APOE ε4 carriers had higher frontal GFAP expression (n=609, β=0.26, p=0.0051), but this association attenuated after adjusting for Aβ (p=0.11). MR supported the causal effect of Aβ on brain GFAP (2SLS β=0.39, p=0.0085). The frontal GFAP– tau association (β=0.10, p=0.012) weakened after controlling for Aβ (p=0.50).

**Conclusion:**

Our study leveraging APOE ε4 as a genetic causal anchor supports that Aβ is at the causal upstream of increased brain GFAP gene expression and elevated plasma GFAP (Figure 1A). By contrast, the neocortical tau – GFAP association is likely confounded by Aβ (Figure 1B‐C).